# TCBIR/CD320: a potential therapeutic target upregulated in endothelial cells and associated with immune cell infiltration in liver hepatocellular carcinoma

**DOI:** 10.1007/s12672-024-01122-w

**Published:** 2024-07-02

**Authors:** Shubin Zhang, Zhongyi Jiang, Pusen Wang, Weihao Jiang, Wei Ding, Lin Zhong

**Affiliations:** 1 Department of Hepatobiliary & Pancreatic Surgery, The First Affiliated Hospital of Shandong Second Medical University, School of Clinical Medicine, Weifang People’s Hospital, Shandong Second Medical University, No. 151 Guangwen Street, Weifang, 261041 Shandong China; 2 Department of General Surgery, The Third People’s Hospital of Shenzhen, The Second Hospital Affiliated of Southern University of Science and Technology, Guangdong, China; 3 Department of General Surgery, Shanghai General Hospital, Shanghai Jiao Tong University School of Medicine, Shanghai, China

**Keywords:** TCBIR/CD320_1_, Liver hepatocellular carcinoma_2_, Prognostic biomarker_3_, Immune Infiltration_4_, Tumor angiogenesis_5_

## Abstract

CD320, which is a transmembrane protein responsible for facilitating the absorption of vitamin B12, plays a key role in this process. However, the relationships between CD320 and immune cell infiltration levels remain unclear, with limited studies investigating the diagnostic and prognostic significance of CD320 in hepatocellular carcinoma. We used various databases, including the TIMER, GEPIA, UALCAN and TCGA databases to investigate the expression levels of CD320 in hepatocellular carcinoma. Subsequently, we analyzed the prognosis of hepatocellular carcinoma patients with different expression levels of CD320. Furthermore, we also performed Western blot, immunohistochemistry, and immunofluorescence analyses to validate the results of the database analysis. Finally, the functions of CD320 in hepatocellular carcinoma were also confirmed via relevant cell experiments and angiogenesis assays. We found that CD320 expression was significantly upregulated in tumor vascular endothelial cells. Moreover, the knockdown of CD320 led to a reduction in angiogenesis in endothelial cells. Increased expression of CD320 was also correlated with a poor prognosis in patients with hepatocellular carcinoma, which suggested that CD320 may be a potential prognostic marker. Finally, TIMER analysis demonstrated that the infiltration of six immune cell types was significantly associated with high expression levels of CD320 in hepatocellular carcinoma. Herein, we demonstrated that CD320 may play an important role in angiogenesis in hepatocellular carcinoma. These findings suggested that CD320 may be a potential clinical prognostic marker and immunotherapy target for hepatocellular carcinoma.

**Supplementary Information:**

The online version contains supplementary material available at 10.1007/s12672-024-01122-w.

## Introduction

As the fourth most common cause of cancer fatality worldwide, the incidence and mortality rates of liver hepatocellular carcinoma (LIHC) are increasing every year [1, 2]. In Eastern and Southeastern Asia, as well as Northern and Southern Africa, the highest incidence rates of LIHC were observed, with approximately 50% of all cases occuring in China [3]. Most patients are in advanced stages when they are diagnosed with LIHC because of the high proliferative potential of the tumor and these patients are unable to undergo surgery [4]. The structures of tumor endothelial cells are distinctly different from those of vascular endothelial cells in healthy tissues in terms of processes such as immunosurveillance evasion and hyperpermeability [5, 6]. Moreover, the abnormal vascular system in tumors leads to a decrease in pH, hypoxia, and reduced drug penetration within the tumor microenvironment (TME) [7, 8]. Based on these findings, a multitude of studies have been performed on tumor vascular endothelial cells. Xu et al. discovered that the deletion of Shp2 in tumor vascular endothelial cells resulted in tumor vascular normalization and decreased the growth efficiency of tumor [9]. Sun and his team discovered that, after inhibiting CD93, the tumor vasculature could be normalized, by enhancing blood flow, and increasing tumor hypoxia, moreover, the anti-tumor response to gemcitabine or fluorouracil was also strengthened [10].

Vitamin B12 (cobalamin, Cbl) plays a key role in the folate recycling process, can form a new complex by combining with transcobalamin (TC), which is known as TC-Cbl [11–13]. CD320, which is also known as transcobalamin receptor (TCblR), is a transmembrane protein that can specifically bind with TC-Cbl and facilitate the uptake of vitamin B12 [14]. According to previous reports, CD320 can be expressed in most cell types, including endothelial cells [15–17]. For example, in intestinal epithelial cells, Cbl can be absorbed via CD320 after combining with TC and released into the portal circulation [18]. In addition, CD320 expression levels were also found to be correlated with the proliferative phase of the cell cycle, with the highest expression levels occuring between 24 and 48 h after cells begin to proliferate, furthermore, CD320 overexpression was also observed in highly proliferative cancers [14]. Another report demonstrated that anti-cancer drugs and toxins can be delivered through CD320 to enhance targeted therapeutic effects [19].

Although the expression of CD320 has been shown to be upregulated in many tumors, its functions in tumor vascular endothelial cells in LIHC remain unclear [20].

## Materials and methods

### Single-cell RNA sequencing analysis

Single-cell RNA sequencing analysis of CD320 expression levels and cellular localization in LIHC was conducted by using the publicly available Database of Tumor Microenvironment in Primary and Relapsed Hepatocellular Carcinoma (https://db.cngb.org/PRHCCdb/), which was generated by Zhongshan Hospital, Fudan University, Shanghai, China[21].

### UALCAN

UALCAN (http://ualcan.path.uab.edu/) is an analytical tool built upon a web-based platform, which was designed for the interpretation of transcriptome data from The Cancer Genome Atlas (TCGA) and MET500 datasets. Using UALCAN, we examined the expression levels of CD320 and the correlations between CD320 and various clinical parameters of LIHC, including patient gender and age, cancer stages, tumor grade, nodal metastasis status, and TP53 mutation status.

### Tumor immune estimation resource (TIMER)

The TIMER interactive web portal (https://cistrome.shinyapps.io/timer/) offers a comprehensive approach to examining the infiltration levels of various immune cells [22]. By performing the ‘‘Diff Exp’’ module, we evaluated CD320 expression levels in different tumor types. Additionally, the ‘‘Gene’’ module was used to investigate the correlation between CD320 and the infiltration of immune cells, including B cells, CD8^+^ cells, CD4^+^ cells, macrophages, and dendritic cells. The ‘‘Correlation’’ module was used to analyze the associations between CD320 expression levels and immune cell gene marker sets. Moreover, Spearman’s correlation and statistical significance were used to examine the relationships between CD320 expression levels and the infiltration levels of immune cells.

### PrognoScan database analysis

The PrognoScan database (http://www.abren.net/PrognoScan/) was utilized to investigate the correlations between CD320 expression levels and survival rates in LIHC patients. Using PrognoScan, we examined the associations between CD320 expression levels and patient prognosis, including encompassing overall survival (OS) and relapse-free survival (RFS), in publicly available cancer microarray datasets. The screening parameters, ‘‘cancer type’’ and ‘‘subtype’’, were set to ‘‘liver cancer’’ and ‘‘liver hepatocellular carcinoma’’ respectively, to identify datasets for inclusion in our study. The results are displayed with hazard ratios (HRs) with 95% confidence intervals (95% CIs) and P or Cox p-values from the log-rank test.

### Gene expression profiling interactive analysis (GEPIA)

GEPIA (http://gepia.cancer-pku.cn/index.html) is a web-based portal that uses the TCGA and GTEx databases to conduct gene expression analysis [23]. In this study, we used TCGA-LIHC datasets to investigate the expression levels of CD320. By selecting TCGA and GTEx data, we assessed CD320 expression levels in both LIHC and normal liver tissues by using the ‘‘Expression DIY’’ module of GEPIA. Furthermore, to explore the relationships between CD320 and PD-1, PD-L1, and CTLA-4, we conducted Spearman's correlation analysis.

### Analysis of CD320-interacting genes and proteins

The CD320 interaction network was constructed by using the GeneMANIA database (http://www.genemania.org), whereas the CD320 protein–protein interaction (PPI) network was delineated through the STRING online database (https://string-db.org/) [24].

### Gene ontology (GO) term and Kyoto Encyclopedia of genes and genomes (KEGG) pathway enrichment analysis

GO and KEGG analyses were subsequently performed to determine the biological functions of CD320 in LIHC. GO analysis was used to investigate the biological processes (BPs), cellular components (CCs), and molecular functions (MFs) associated with CD320. The potential mechanisms of CD320 were analyzed via KEGG enrichment analysis. The R package Cluster Profiler was used to conduct GO and KEGG analyses.

### Kaplan–Meier plotter database analysis

Kaplan–Meier plotter (http://kmplot.com), that can be used to analyze gene expression data and survival information, is an online database. Herein, we used it to analyze the prognostic value of CD320 in LIHC tissues, including overall survival (OS), progression-free survival (PFS) and disease-free survival (DSS) and so on.

### Tumor samples and collection

All six LIHC tissues and paired non-tumor liver tissues from humans were collected at Shanghai General Hospital from January 2017 to January 2023. All of the patients signed informed consent and they underwent surgery for the first time without previous radiotherapy or chemotherapy. This study was approved by the ethics committee of Shanghai General Hospital (2021KSQ341).

### Western blotting, immunofluorescence (IF) staining, and immunohistochemistry (IHC)

For Western blotting, we employed SDS-PAGE to isolate total proteins from human LIHC and normal liver tissues, followed by the transfer of these proteins onto polyvinylidene fluoride (PVDF) membranes (Millipore, IPVH00005). The primary antibodies utilized in the WB procedure included anti-rabbit CD320 (Proteintech, 10,343-1-AP) and anti-mouse β-actin, GB12001-100).

In the IF procedure, we collected six human tissue samples and preserved them in formalin. These tissues were then embedded in paraffin and sliced using a microtome. Subsequently, the slides were dewaxed, hydrated, and incubated with primary antibodies at 4 ℃ overnight. Subsequently, the slides were washed and incubated with secondary antibodies at room temperature for an hour. The primary antibodies used for IF were anti-rabbit CD320 (Proteintech, 10,343-1-AP) and anti-mouse CD31 (Invitrogen, 14-0311-82).

For IHC, we selected six groups of samples, including LIHC and non-tumor tissues. After fixation in formalin, the samples were subsequently embedded in paraffin and sliced. The slices were mounted on slides and subjected to dewaxing and antigen retrieval. Subsequently, we used 3% hydrogen peroxide to quench endogenous peroxidase activity and then washed the slices with PBS three times. The sections were then incubated with an anti-rabbit CD320 antibody (Proteintech, 10,343-1-AP) overnight at 4 ℃. Finally, the slices were washed three times with PBS and incubated them with secondary antibodies for approximately 30 min. The results of these samples were evaluated by two pathologists who did not konw each other.

### Cell lines

Human Umbilical Vein Endothelial Cell (HUVEC), Hep3B, Huh7, and LM3 cell lines were cultured in Dulbecco’s Modified Eagle’s Medium (DMEM) (Gibco, Grand Island, NY, USA), supplemented with 10% fetal bovine serum (Gibco) and 1% Penic-Streptomycin Solution (Gibco). All of the cells were incubated at 37 °C with 5% CO_2_ environment.

### Conditioned medium (CM)

The Hep3B, Huh7, and LM3 cell lines were cultured in DMEM supplemented with 10% fetal bovine serum and 1% Penicillin–Streptomycin Solution. After reaching a cell density of 80%, the culture medium was replaced with DMEM without fetal bovine serum. After incubating for 24 h, the supernatant was collected. Subsequently, the supernatant was centrifuged at 3500 rpm/min, filtered through 0.22 μm filters, and stored at – 20 ℃.

### Angiogenesis experiment

Angiogenesis experiments were performed to investigate the functions of genes in endothelial cells. First, we thawed the matrix, which was purchased from Yeasen Company (CeturegelTM Matrix GFR, Phenol Red-Free, LDEV-Free, 4086ES08), and added it to 96-well plates at a volume of 50 μl per well. Subsequently, the matrix was incubated at 37 for 30 min. When the cell density reached 80%, they were seeded onto the matrix surface at a concentration of 4000 cells per well and supplemented with DMEM (100 μl) supplemented with 10% fetal bovine serum. Finally, we captured images and observed the number of chambers at 2 h, 5 h, and 8 h to study the function of CD320 in endothelial cells.

### Cell transfection

The Short hairpin RNA (shRNA) specific for CD320 (sh-CD320) and the negative control sequence (sh-NC) were obtained from Zorin (Zin, Shanghai, China). The target sequences of sh-CD320 and sh-NC were 5ʹ—AGTGCAGGATTGAGCCATGTA—3ʹ and 5ʹ—TTCTCCGAACGTGTCACGT—3ʹ, respectively. HUVECs were transfected with shRNA following the manufacturer's protocol.

### Statistical analysis

The results of Kaplan–Meier plotter and GEPIA are showed with HR and P or Cox P-values from a log-rank test. The correlation of CD320 gene expression was explored by Spearman’s correlation and statistical significance. The P-values < 0.05 were considered statistically significant for all statistical analyses.

## Results

### Pancancer analysis of CD320 expression

**Fig. 1:**
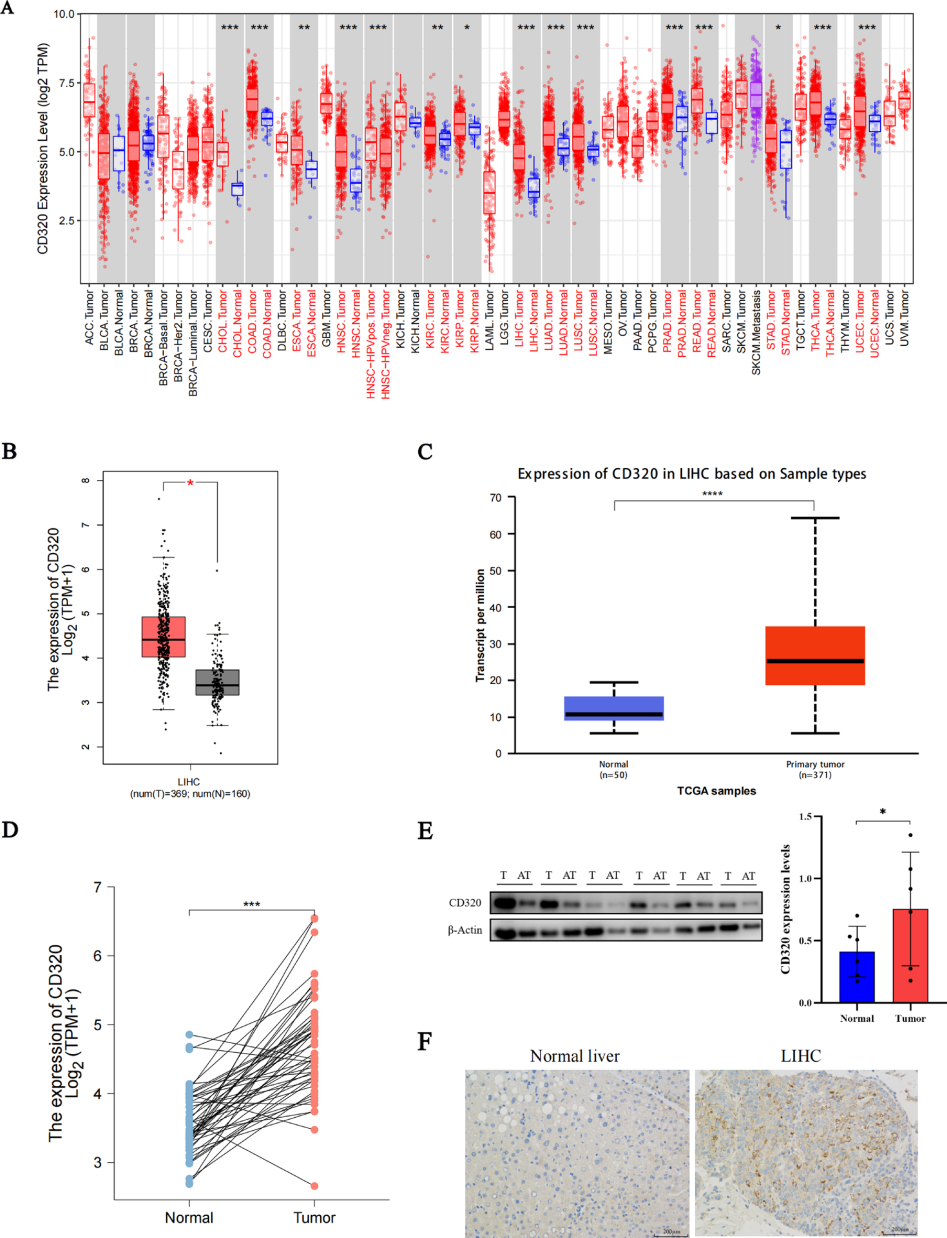
Expression levels of CD320 in different cancers. **A** Expression levels of CD320 were found to be upregulated across various types of cancer. **B** CD320 expression levels were elevated in LIHC compared with normal liver tissues, as corroborated by the GEPIA database. **C** The increased expression levels of CD320 were observed in LIHC tissues by the UALCAN database. **D** The upregulated expression level of CD320 was observed in LIHC from 50 pairs of tumor tissues and matched adjacent tissues in the TCGA database. **E** Expression of CD320 in LIHC tissues was higher than in paired normal liver tissues by WB. **F** Elevated expression of CD320 was detected in LIHC tissues by employing IHC. *p < 0.05, **p < 0.01, ***p < 0.001

By using the TIMER database we discovered that the mRNA expression levels of CD320 were upregulated in many tumors, including cholangiocarcinoma, colon adenocarcinoma, esophageal carcinoma, LIHC, head and neck squamous cell carcinoma, lung squamous cell carcinoma, head and neck squamous cell carcinoma-HPV positive, kidney renal clear cell carcinoma, kidney renal papillary cell carcinoma, lung adenocarcinoma, prostate adenocarcinoma, rectum adenocarcinoma, stomach adenocarcinoma, thyroid carcinoma, and uterine corpus endometrial carcinoma, compared to the corresponding non-tumor tissues **(**Fig. 1A**)**. Furthermore, we observed that the mRNA expression level of CD320 in LIHC tissues was greater than in non-tumor tissues **(**Fig. 1B, C**)**. A significant increase in CD320 expression, compared with that in the respective normal liver tissues, was also detected in 50 pairs of LIHC tumor samples **(**Fig. 1D**)**.

We further examined the protein expression levels of CD320 in LIHC tissues and adjacent non-tumor tissues. The results showed that CD320 was significantly upregulated in LIHC tissues **(**Fig. 1E**)**. IHC showed an increase in CD320 protein expression in LIHC tissues **(**Fig. 1F**)**.

The present findings indicated significant upregulation of CD320 mRNA and protein expression in LIHC, thus suggesting that CD320 may play a crucial role in the oncogenesis and progression of LIHC.

### Clinical parameters and CD320 expression levels in LIHC Patients

**Fig. 2:**
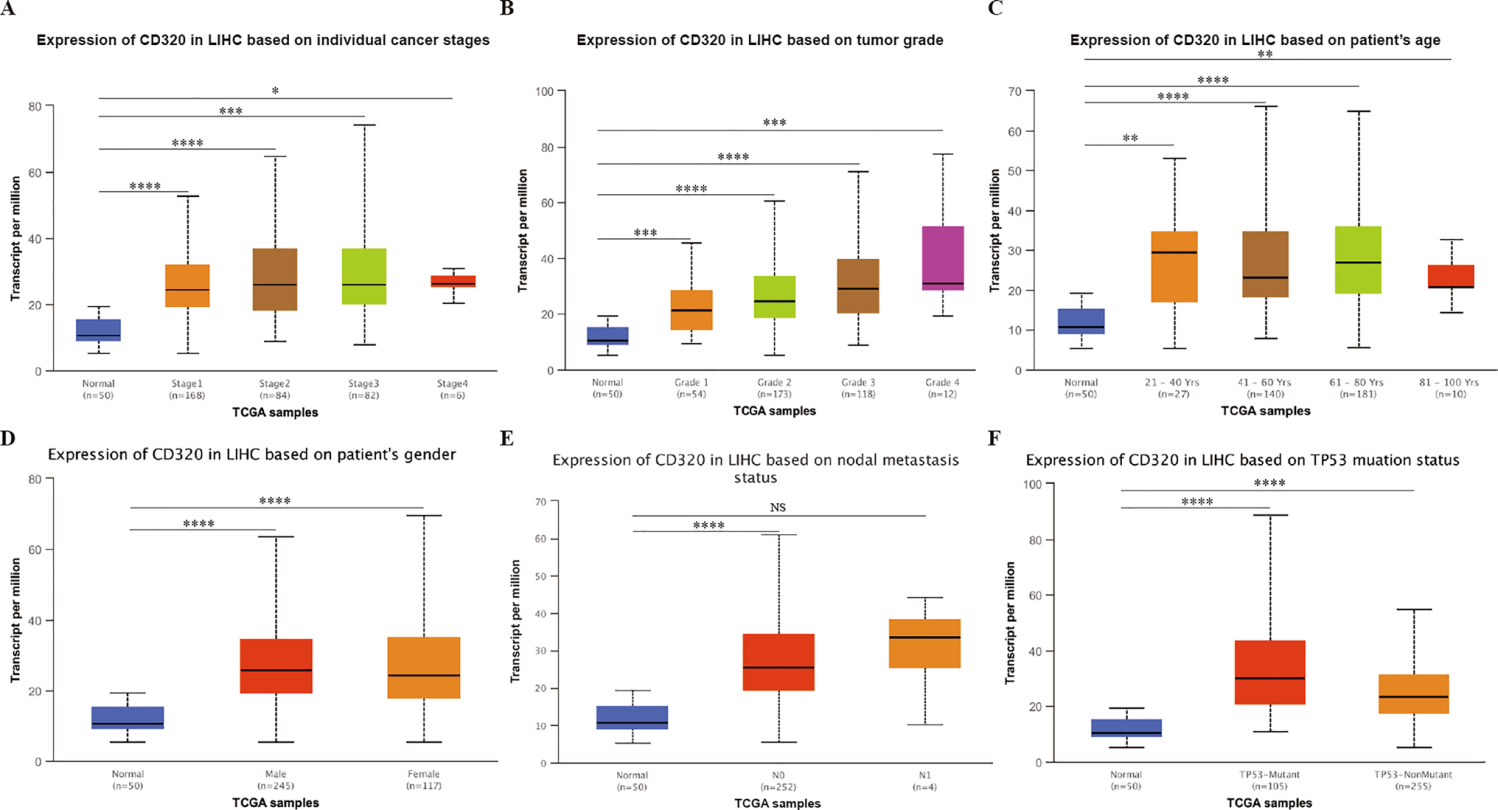
CD320 expression levels between different groups of patients with LIHC based on clinical parameters. The findings demonstrated a significant correlation between CD320 protein expression levels and cancer stages **A**, tumor grades **B**, age **C**, gender **D**, nodal metastasis status **E**, and TP53 mutation status **F** in LIHC. N0: no regional lymph node metastasis; N1: metastases in 1 to 3 axillary lymph nodes. *p < 0.05, **p < 0.01, ***p < 0.001, ****p < 0.0001

After observing that CD320 mRNA expression was upregulated in LIHC, we investigated the protein expression levels of CD320 among various patients who were diagnosed with LIHC by using the UALCAN online tool and TCGA database, based on distinct clinical parameters. In the context of cancer staging, we observed that in patients with LIHC classified as stage 1, 2, 3, and 4, the CD320 expression levels in tumor tissues were elevated compared to those in the corresponding non-tumor tissues **(**Fig. 2A**)**. Based on tumor grade, significant increases in CD320 expression were observed in tumor of grades 1, 2, 3, and 4 **(**Fig. 2B**)**. According to age, we divided patients who suffered LIHC into four groups (21–40 years, 41–60 years, and 61–100 years in LIHC) and observed that CD320 expression was upregulated in each group **(**Fig. 2C**)**. In terms of gender, there was no significant difference in CD320 expression between males and females diagnosed with LIHC **(**Fig. 2D**)**. Regarding the nodal metastasis status, a significant increase in CD320 expression was observed in patients with LIHC classified as N0 **(**Fig. 2E**)**. However, there was no significant difference in CD320 expression between tumor tissues and adjacent non-tumor tissues in patients with LIHC classified N1 **(**Fig. 2E**)**. Furthermore, the upregulation of CD320 expression was also observed in patients diagnosed with LIHC, compared with their corresponding non-tumor tissues, both in those classified as TP53-Mutant and TP53-NonMutant **(**Fig. 2F**)**.

### High CD320 expression is associated with poor prognosis in patients with LIHC

**Fig. 3:**
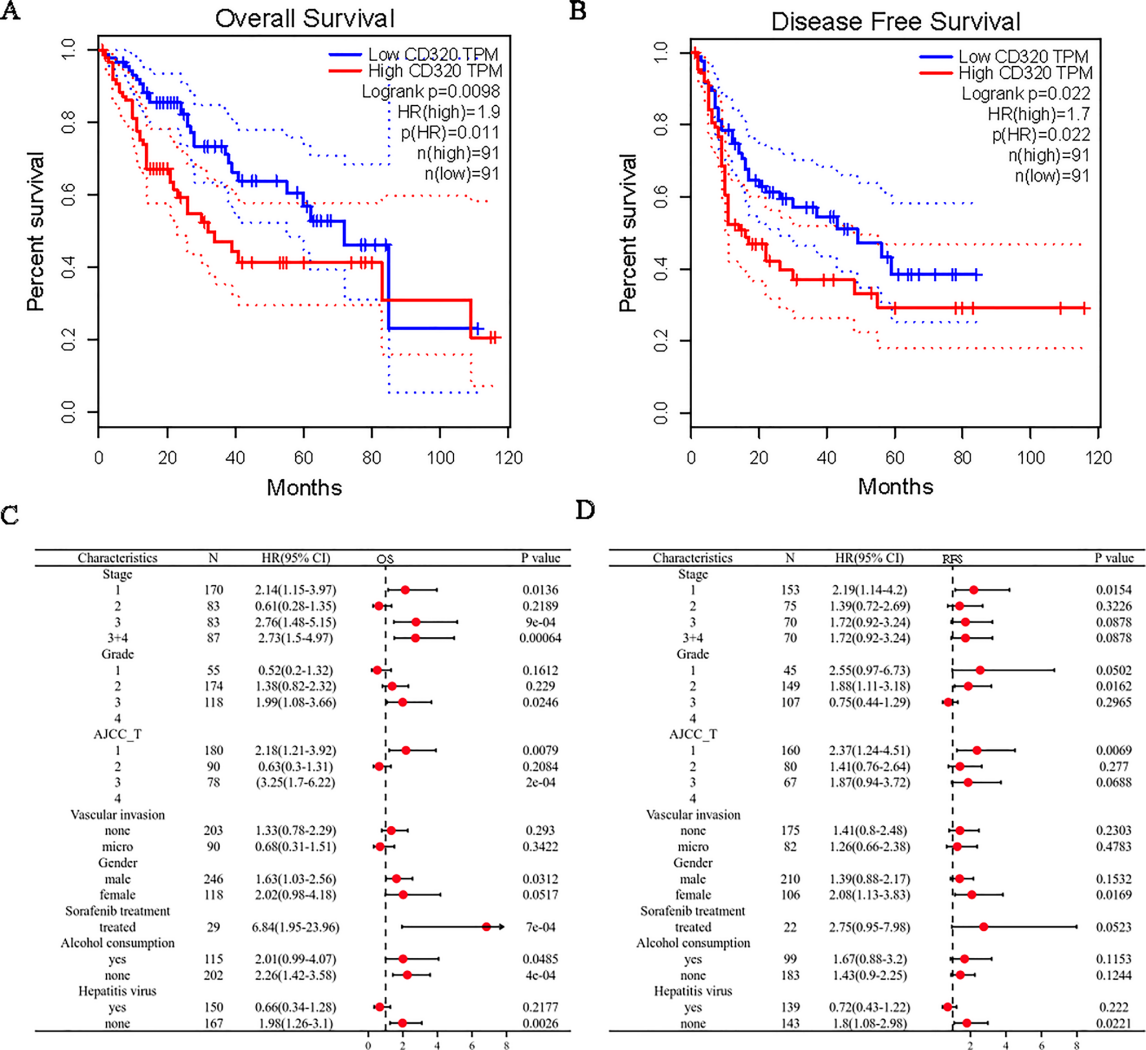
Prognostic values of CD320 in LIHC by survival curses. Patients exhibiting high CD320 expression levels demonstrated a poorer OS **A** and DFS **B** based on the GEPIA. **C**, **D** The associations between CD320 expression levels and various clinicopathological parameters in LIHC were demonstrated using forest plots

We further examined the prognostic significance of CD320 in our study. The OS and disease-free survival (DFS) of patients with high CD320 expression were poor **(**Fig. 3A, B**)**. An increase in CD320 expression was strongly associated with poor disease-specific survival (DSS) in patients with LIHC, but not with progression-free survival (PFS) or RFS (Supplementary Fig. 2A, B, C).

These findings preliminarily indicated that CD320 could be regarded as a potential prognostic marker for patients with LIHC.

### Verifying the prognostic value of CD320 on the basis of various clinicopathological features

We examined the correlations between CD320 mRNA expression levels and clinical characteristics using the Kaplan‒Meier plotter, to further describe the prognostic value and potential mechanisms of CD320 expression in LIHC more comprehensively. The results suggested that high CD320 expression was significantly correlated with poor OS in stage 1, 3, and 4 LIHC patients, but not in stage 2 patients **(**Fig. 3C**)**. And we observed that CD320 expression was only negatively correlated with superior RFS in stage 1 **(**Fig. 3D**)**. Moreover, the upregulation of CD320 expression was also found to be associated with poor OS in patients with grade 3 tumors and with poor RFS in patients with grade 2 tumors **(**Fig. 3C, D**)**. According to the classification of the American Joint Committee on Cancer (AJCC), high CD320 expression was significantly correlated with inferior OS in stage T-1 and T-3 patients and poor RFS in stage T-1 patients with LIHC **(**Fig. 3C, D**)**. In addition, the increase in CD320 was correlated with inferior OS in male LIHC patients and poor RFS in female LIHC patients **(**Fig. 3C, D**)**. In the cohort of patients who had undergone sorafenib treatment, we observed a significant inverse correlation between high CD320 expression and OS **(**Fig. 3C**)**. Furthermore, a notable correlation was detected between high CD320 expression and decreased OS in patients who did and did not consume alcohol **(**Fig. 3C**)**. Ultimately, CD320 expression was also negatively associated with OS and RFS in patients without viral infection **(**Fig. 3C, D**)**.

These findings suggested that CD320 has potential prognostic value for patients suffering from LIHC.

### Verification of the interacting protein, gene and genetic variation of CD320

**Fig. 4:**
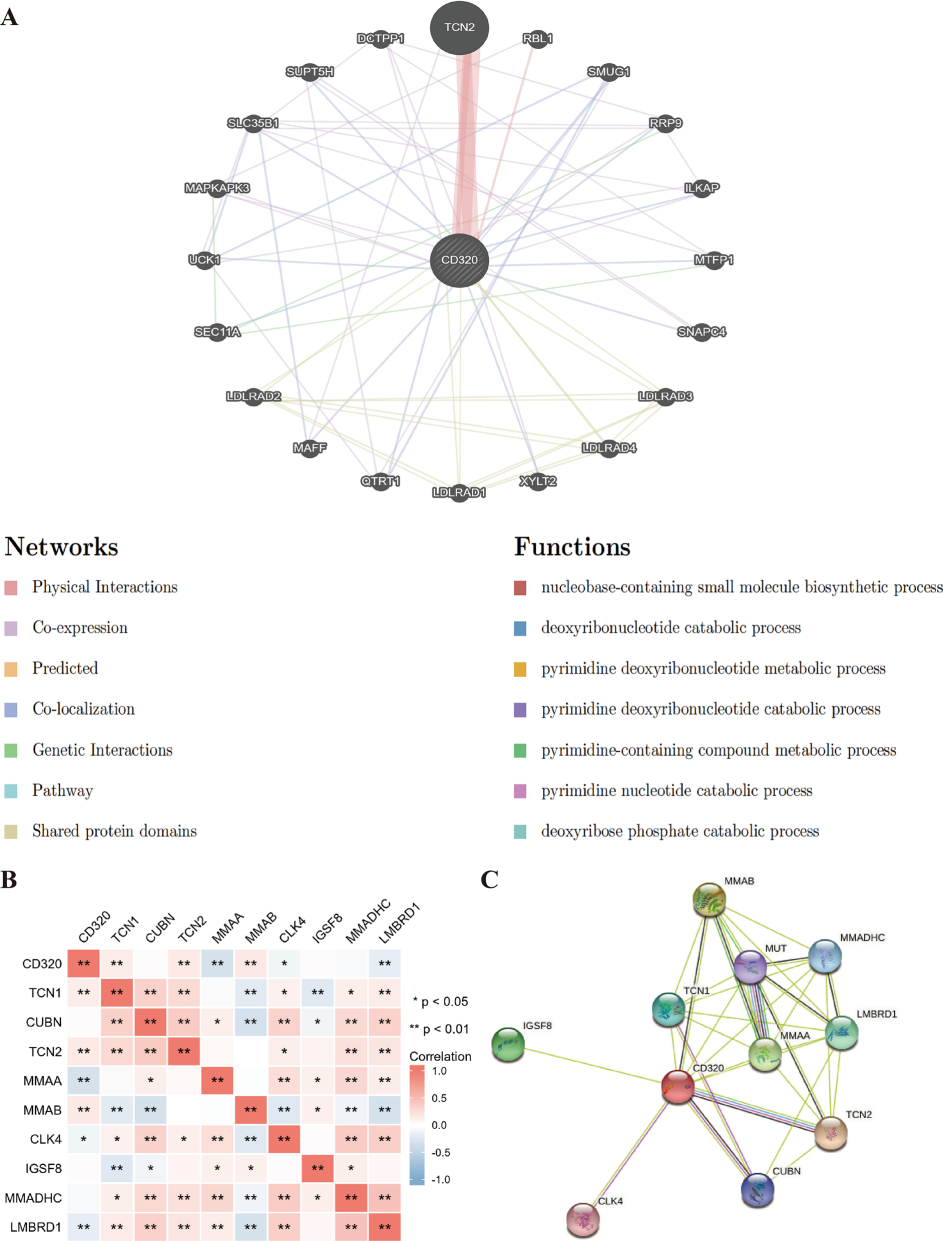
The gene–gene interaction and protein–protein interaction network of CD320 in LIHC. **A** The analysis of GeneMania identified the top 20 most frequently mutated genes in correlation with CD320, within the gene–gene interaction network. **B** The relation between CD320 and Vitamin B12 metabolism-related genes in LIHC was visualized by a heatmap. **C** The construction of the PPI network for CD320 was accomplished using STRING

In addition to investigating the potential prognostic value of CD320, we also sought to elucidate the mechanism of CD320 in LIHC. By performing GeneMania, we constructed a gene interaction network for CD320, along with the mutation of neighboring genes. We identified the top 20 frequently mutated genes associated with CD320, including TCN2, RBL1, and SMUG1 **(**Fig. 4A**)**. The functional analysis showed a striking association between these top 20 genes and the biosynthetic process of nucleobase-containing small molecules **(**Fig. 4A**)**. The correlation between CD320 and vitamin B12 metabolism-related genes in LIHC was also analyzed, and the heatmap revealed a significant positive correlation with TCN1, TCN2, and MMAB but a negative correlation with MMAA, CLK4, and LMBRD1 **(**Fig. 4B**)**. We further constructed a PPI network for CD320 by using the STRING database, which consisted of 32 edges and 11 nodes, including TCN2, CUBN, and MMAA **(**Fig. 4C**)**.

### KEGG and go pathway analysis of CD320 and its co-expressed genes in LIHC

**Fig. 5:**
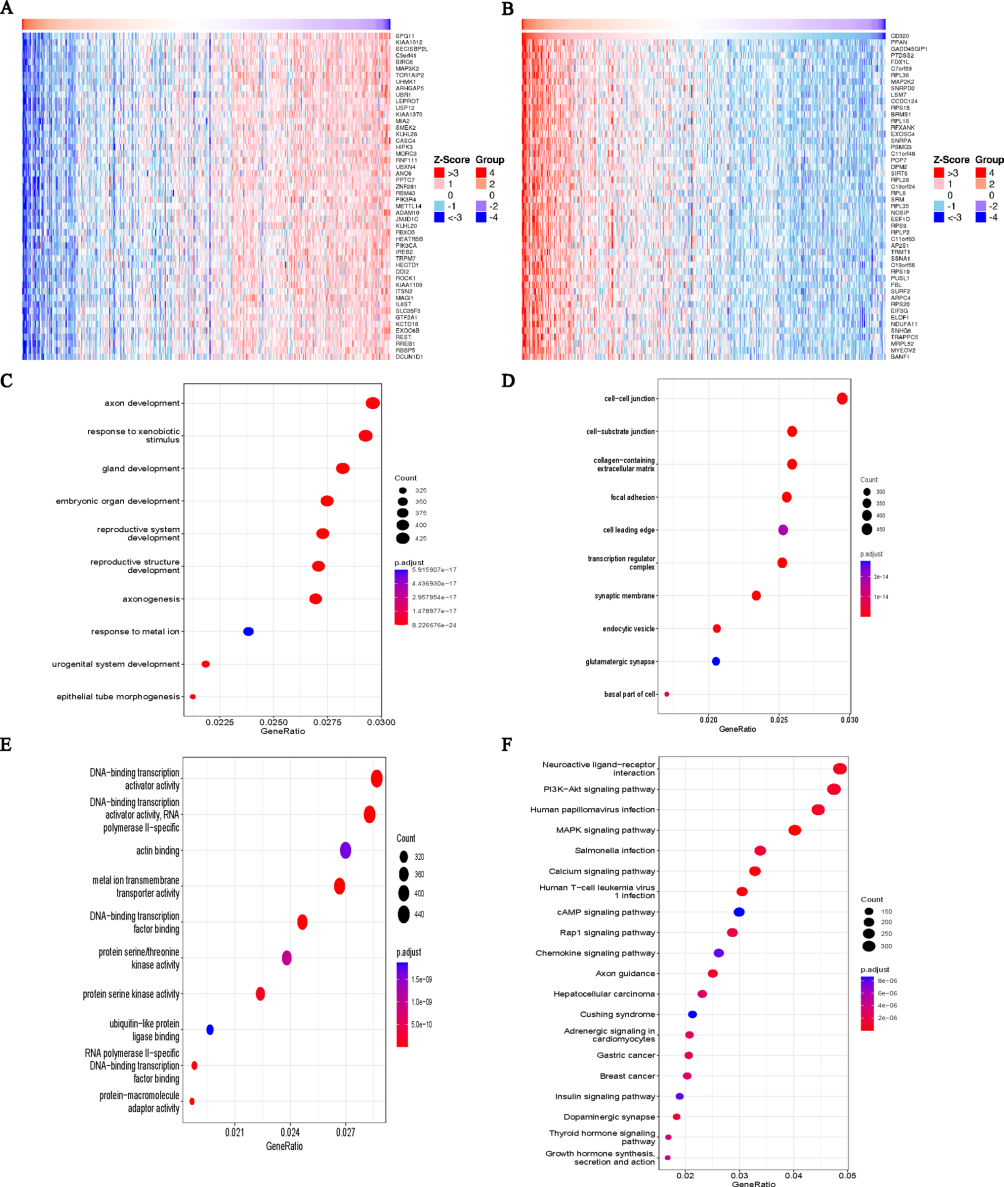
The gene associated with CD320 and functional enrichment analysis of CD320 in LIHC. **A**, **B** Heatmap analysis revealed the top 50 genes, both negatively and positively associated with CD320 in LIHC, respectively. **C**, **D**, **E** Top 10 enrichment terms in BP, CC, and MF categories in LIHC. **F** Top 20 KEGG enrichment pathways in LIHC

Analysis of the TCGA database showed the top 50 genes that were positively or negatively associated with CD320 in LIHC (Fig. 5A, B). The 20 most significant terms derived from BP, CC, and MF enrichment analysis, as well as KEGG pathway analysis are presented in (Fig. 5C–F). The findings suggested that CD320 was prominently involved in processes associated with organ development, including axon development, response to xenobiotic stimulus, gland development, and embryonic organ development (Fig. 5C). The enriched CC terms were cell–cell junction, cell-substrate junction, and focal adhesion (Fig. 5D). The enriched MFs included DNA-binding transcription activator activity, actin binding, and metal ion transmembrane transporter activity (Fig. 5E). Furthermore, pathway analysis showed that CD320 was strongly associated with the PI3K-Akt signaling pathway, human papillomavirus infection, the MAPK signaling pathway, and Salmonella infection (Fig. 5F).

### Correlation analysis of CD320 expression and infiltration of immune cells

**Fig. 6:**
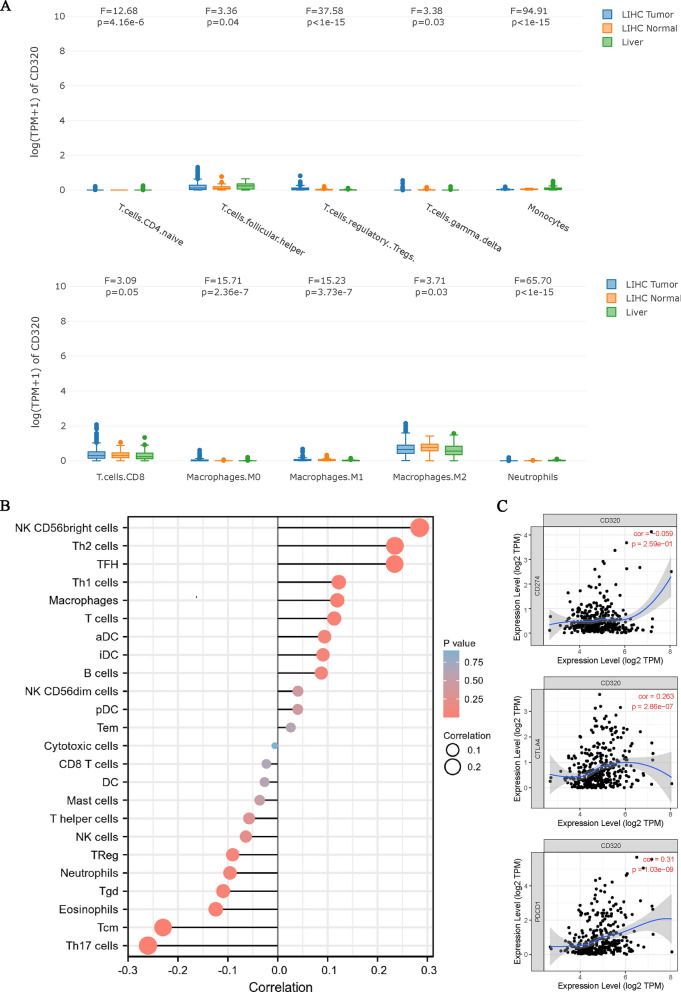
The association between CD320 and immune cell infiltration. **A** CD320 was positively correlated with the infiltration of different immune cells by using the GEPIA database. **B** Positive and negative correlations between CD320 and immune cells in OS were shown. **C** CD320 was significantly correlated with parts of immune checkpoints through using the TIMER

The relationships between the expression of CD320 and the infiltration of six types of immune cells were also investigated. CD320 exhibited significant interactions with CD4^+^ T cells, T follicular helper (TFH) cells, regulatory T cells (Tregs), Tγδ cells, monocytes, macrophages (including M0, M1, and M2), and neutrophils **(**Fig. 6A**)**. We further evaluated the correlations between CD320 expression and the infiltration levels of six types of immune cells **(**Fig. 6B**)**. The results showed the positive correlations between CD320 and natural killer (NK) CD56bright cells, T helper 2 (Th2) cells, and TFH cells **(**Fig. 6B**)**. Conversely, negative associations were also observed between CD320 and Th17 cells, central memory T (TCM) cells, and eosinophils **(**Fig. 6B**)**. Moreover, we observed significant correlations between CD320 and immune checkpoint proteins, including CTLA4 and PDCD1 **(**Fig. 6C**)**. In addition, we employed CIBERSORT to examine the relationship between CD320 and immune infiltration and focused on the impact of CD320 on the TME. The findings demonstrated positive correlations between CD320 and the infiltration levels of macrophages, CD56bright cells, T cells, T follicular helper cells, Th1 cells, and Th2 cells, but negative correlations with the infiltration levels of eosinophils, central memory T cells, and Tγδ Th17 cells (Supplementary Fig. 3). These findings verified that CD320 significantly contributes to immune cell infiltration in LIHC.

### Analysis of the association between prognosis and CD320 expression in LIHC patients based on immune cell infiltration

**Fig. 7:**
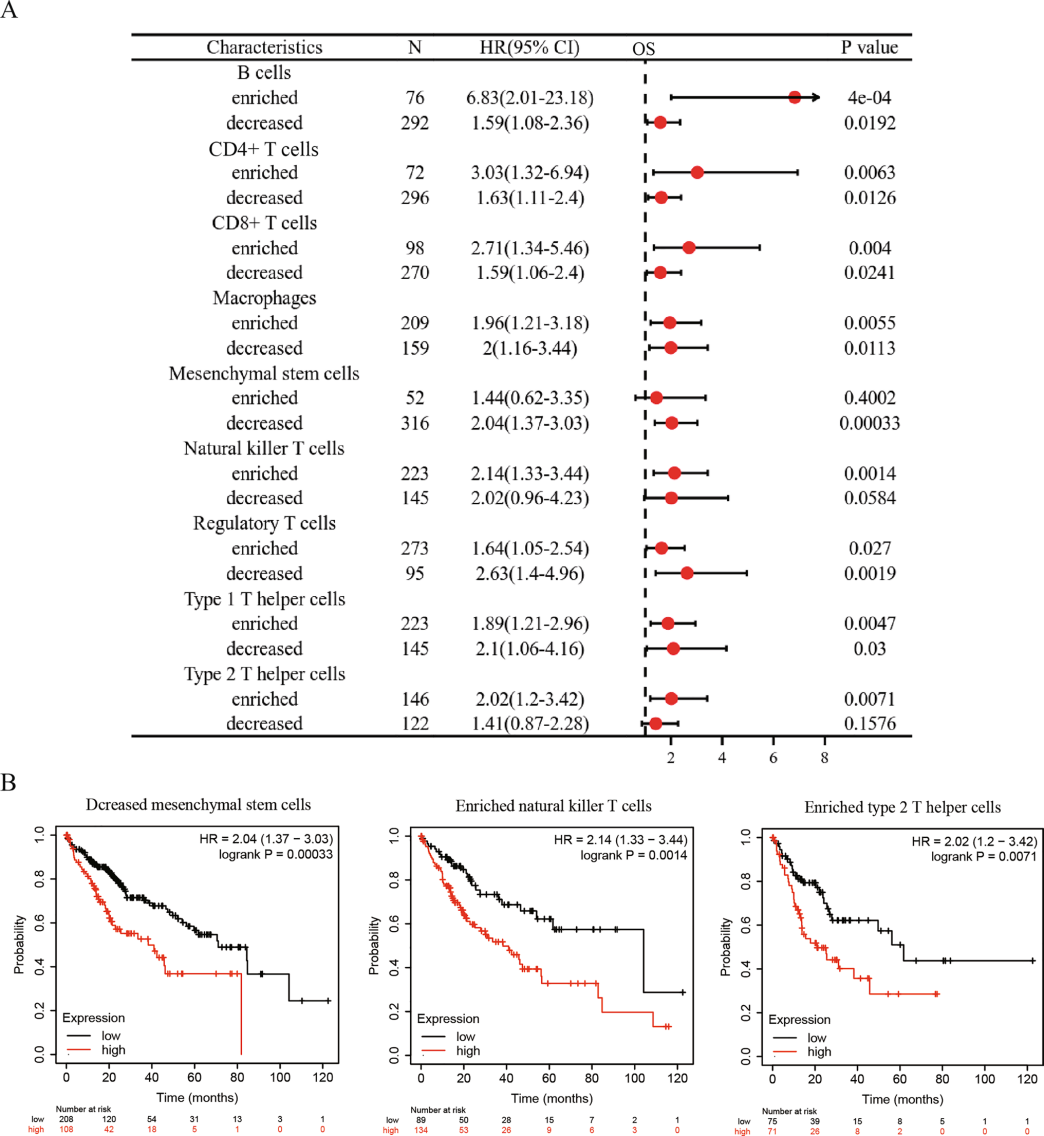
Prognostic prediction of CD320 expression based on immune cells. **A** According to various immune cell subtypes, the prognostic values of CD320 for the OS of patients with LIHC were depicted in a forest plot. **B** The associations between CD320 expression levels and OS in different subtypes of immune cells in patients with LIHC by the Kaplan–Meier plotter

We previously reported of significant associations between high CD320 expression and adverse outcomes, as well as immune cell infiltration. Therefore, we investigated the potential impact of CD320 expression on immune infiltration. The findings suggested that high expression of CD320 with abundant infiltration of B cells, CD4^+^ memory T cells, CD8^+^ T cells, macrophages, natural killer T cells, Tregs, and type 12 T helper cells was negatively correlated with a favor prognosis in patients diagnosed with LIHC **(**Fig. 7A, B**)**. In contrast, a significant increase in CD320 expression and a decrease in the infiltration of B cells, CD4^+^ memory T cells, CD8^+^ T cells, macrophages, mesenchymal stem cells, regulatory T cells, and type 1 T helper cells were found to be associated with poor prognosis in patients with LIHC **(**Fig. 7A, B**)**.

These findings indicated that CD320 could influence the prognosis of LIHC patients by affecting the infiltration of immune cells.

### Location of CD320 in tumors

**Fig. 8:**
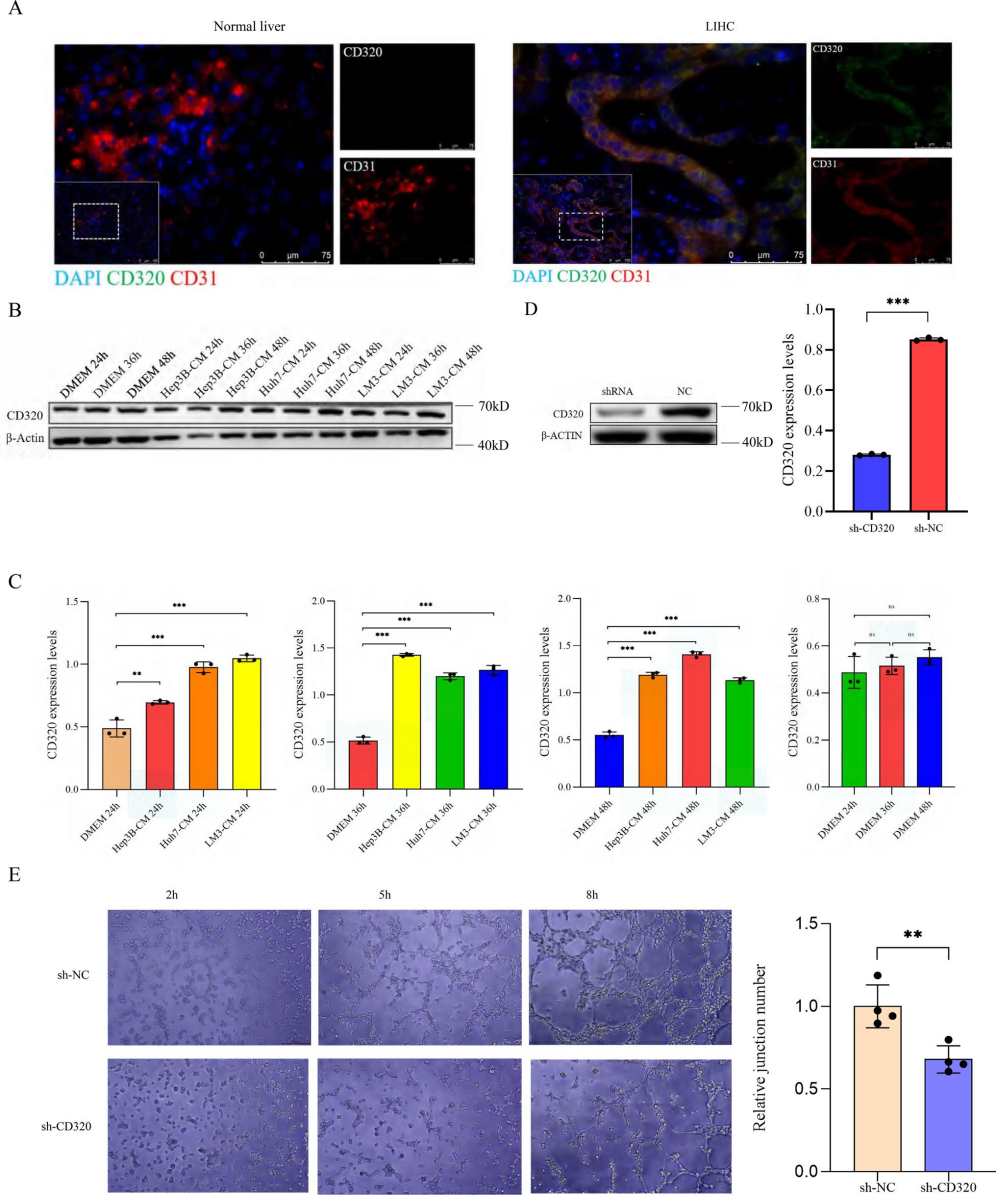
Effect of CD320 expression level on tumor angiogenesis in LIHC. **A** The results showed that CD320 was co-localized with CD31 in LIHC by IF. **B** CD320 expression levels were increased in Hep3B, Huh7, and LM3 cell lines after being stimulated by CM. **C** The results of WB showed that the level of CD320 expression was knocked down in HUVECs. **D** The statistical charts showed that CD320 expression levels were increased significantly in Hep3B, Huh7, and LM3 cell lines that were stimulated by using CM. **E** The capacities of tubes generated were reduced in CD320 knockdown groups compared with control groups. *p < 0.05, **p < 0.01, ***p < 0.001

To further elucidate the functions of CD320 in LIHC, we conducted a series of experiments to investigate CD320. Single-cell RNA sequencing analysis of LIHC patient tissues revealed increased expression levels of CD320 in tumor endothelial cells compared with those in corresponding non-tumor tissues (Supplementary Fig. 1), and CD320 was colocalized with CD31 (Supplementary Fig. 1E, F). We observed that CD320 was colocalized with CD31, as demonstrated by IF analysis, and our findings also indicated that the upregulation of CD320 was primarily observed in tumor endothelial cells **(**Fig. 8A**)**. Subsequently, we replaced DMEM with three distinct CMs from HUVECs, thereby creating a simulated tumor microenvironment to promote the transformation of HUVECs into tumor endothelial cells, moreover, we harvested cells and isolated proteins after 24, 36, and 48 h. WB analysis demonstrated a significant increase in CD320 expression in the experimental group compared to that in the control group in which only DMEM was used for cell culture, and no significant differences were observed within the control group (Fig. 8B, C). These results suggested that CD320 was mainly upregulated in tumor endothelial cells. Additionally, we successfully knocked down CD320 expression in HUVECs, as confirmed by the results of WB (Fig. 8D). We further conducted angiogenesis experiments and demonstrated a significant reduction in the capacity for blood vessel formation in the sh-CD320 group compared to the NC group (Fig. 8E).

In summary, we found that CD320 was predominantly upregulated in tumor vascular endothelial cells and that this alteration affected the capacity of endothelial cells to form blood vessels.

## Discussion

LIHC is the fourth leading cause of cancer-related deaths throughout the world, with increasing mortality and incidence rates [1, 2]. Approximately 80% of hepatitis cases worldwide are attributed to hepatitis B virus (HBV) and hepatitis C virus (HCV) [25]. The majority of patients are at advanced stages when diagnosed with LIHC, thus resulting in limited treatment modalities and a dismal prognosis [26, 27]. Alpha-fetoprotein (AFP) is the predominant serum biomarker for liver cancer screening in LIHC patients. However, its limited sensitivity and specificity constrain its clinical utility [28, 29]. Agopian et al. reported that a significant proportion of LIHC patients exhibited low serum AFP levels in their study, which reported that 31.3% of non-AFP-producing tumors [30]. Thus, the exploration of novel biomarkers in LIHC is crucial for enhancing diagnostic and prognostic accuracy, thereby improving patient outcomes.

The expression levels of CD320 are elevated in several tumors and some studies have investigated CD320-mediated targeted therapies for tumors [20, 31]. However, the role of CD320 and its regulatory mechanisms in tumor endothelial cells within LIHC are still unclear. In this study, we found that CD320 may play an important role in tumor growth and metastasis by impacting endothelium development and angiogenesis. Analysis of the databases indicated that CD320 expression levels were greater in LIHC tissues than in non-tumor tissues, and the results of WB, IHC, and single-cell sequencing experiments confirmed this finding. CD320 expression was also significantly correlated with age, sex, cancer stage, tumor grade, nodal metastasis status, and TP53 expression in patients who suffer from LIHC, and patients with high CD320 expression in LIHC exhibited a dismal survival rate in our study. This indicates that CD320 may be a potential independent prognostic biomarker in patients with LIHC. Although there are some reports about CD320-mediated targeted drugs for treating some cancers [19, 31–33], there are no drugs that block or downregulate the CD320 protein to treat patients suffering from LIHC. In addition, a significant correlation was observed between the upregulation of CD320 expression and inferior OS, PFS, and DSS in patients with LIHC, and this association was particularly prominent in patients treated with sorafenib, who exhibited poorer OS than in those with lower CD320 expression levels. However, the underlying mechanism linking CD320 and sorafenib remains to be elucidated.

The findings of our research corroborate the notion that CD320 is intricately linked to tumor angiogenesis, as our angiogenesis experiments demonstrated a significant reduction in the angiogenesis capacity of endothelial cells following the knockdown of CD320. A systematically constructed gene–gene interaction network that was constructed by using GeneMania revealed the top 20 most frequently altered genes associated with high CD320 expression. Among these genes, RB transcriptional corepressor like 1 (Rbl1) was found to promote subretinal angiomatous proliferation and hemangioblastoma in mice upon loss [34]. When the retinoblastoma tumor suppressor Rb1/Rbl1 is absent in the retina, the development of retinoblastoma is inhibited. However, subretinal angiomatous proliferation (RAP) and retinal capillary hemangioblastoma (RCH) are induced following the removal of Von Hippel–Lindau (Vhl) [34]. In conjunction with our findings, the potential inhibitory effects of CD320 on Rbl1, through an unidentified mechanism may also repress tumor vascular growth.

We also conducted a subgroup analysis focusing on immune cells. The findings revealed that a high expression level of CD320, accompanied by increased infiltration of CD4 + T cells, TFH cells, Tregs, gamma delta cells, monocytes, macrophages (including M0, M1, and M2), and neutrophils, was associated with a poor prognosis in patients with LIHC. Blockage of the CD320 signaling pathway could sensitize tumors to immunotherapy, thereby enhancing the efficacy of tumor immunotherapy. Furthermore, increased expression of CD320 is positively correlated with several well-known immune checkpoints, such as PDCD1 and CTLA4, during immunotherapy. These immune checkpoints have been reported to exert their effects via either the PI3K-Akt signaling pathway or the MAPK signaling pathway [35–37]. Our analysis of the top 20 KEGG enrichment pathways in LIHC demonstrated a significant association between high CD320 expression and the PI3K-Akt signaling pathway, and MAPK signaling pathway, among others. This finding suggests that CD320 potentially influences tumor angiogenesis through these classical signaling pathways.

### Shortcomings

In our study, we only collected tumor and non-tumor samples from six patients who were diagnosed LIHC, and need to collect more samples to verify our results. Besides, the analysis of stage 1–3 in LIHC based on individual cancer stages was also missing and we need to perform analysis of this section to explore the association between CD320 and the clinical progression of LIHC in the future study.

## Conclusion

In summary, we discovered that CD320 was upregulated in tumor vascular endothelial cells in LIHC. We also found that CD320 may contribute to tumor progression and metastasis by regulating immune cell infiltration within the tumor-immune microenvironment and is a potential novel immunotherapy target for LIHC. In addition, CD320 also exhibits potential prognostic value and is expected to be a potential immunotherapy target.

### Supplementary Information

Additional file 1 (TIF 21496 KB)

Additional file 2 (TIF 1628 KB)

Additional file 3 (TIF 9191 KB)

## Data Availability

The original contributions presented in the study are included in the article/Supplementary Materials. Further inquiries can be directed to the corresponding authors.

## Abbreviations

AFP: Alpha-fetoprotein
AJCC: American Joint Committee on Cancer
BPs: Biological processes
Cbl: Vitamin B12
CCs: Cellular components
CM: Conditioned medium
DFS: Disease-free survival
DMEM: Dulbecco’s Modified Eagle’s Medium
DSS: Disease-specific survival
GEPIA: Gene expression profiling interactive analysis
GO: Gene ontology
HBV: Hepatitis B virus
HCV: Hepatitis C
HR: Hazard ratio
HUVEC: Human umbilical vein endothelial cell
IF: Immunofluorescence
IHC: Immunohistochemistry
KEGG: Kyoto Encyclopedia of Genes and Genomes
LIHC: Liver hepatocellular carcinoma
MFS: Molecular functions
NK: Natural killer
OS: Overall survival
PFS: Progression-free survival
PVDF: Polyvinylidene fluoride
RAP: Subretinal angiomatous proliferation
Rbl1: RB transcriptional corepressor like 1
RCH: Retinal capillary hemangioblastoma
RFS: Relapse-free survival
shRNA: Short hairpin RNA
TC: Transcobalamin
TCblR: Transcobalamin receptor
TCGA: The Cancer Genome Atlas
TCM: Central memory T cells
TFH: T follicular helper cells
Th2: T helper 2 cell
TIMER: Tumor Immune Estimation Resource
TME: Tumor microenvironment
Tregs: Regulatory T cells
