# Supplementary figures and images for: TCBIR/CD320: a potential therapeutic target upregulated in endothelial cells and associated with immune cell infiltration in liver hepatocellular carcinoma
Source: Discov Oncol. 2024 Jul 2;15:255. doi: 10.1007/s12672-024-01122-w (PMC11219609; doi:10.1007/s12672-024-01122-w)

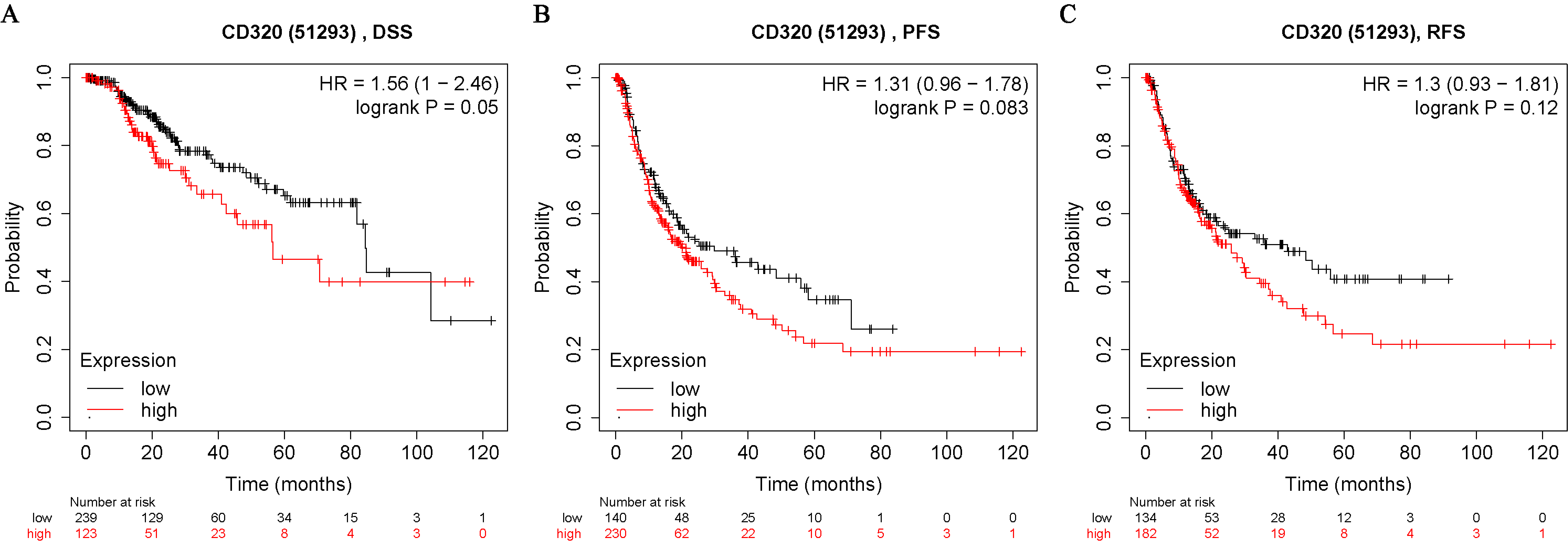

Supplement: Supplementary file 2 — Additional file 2 (TIF 1628 KB) [file 12672_2024_1122_MOESM2_ESM.tif]

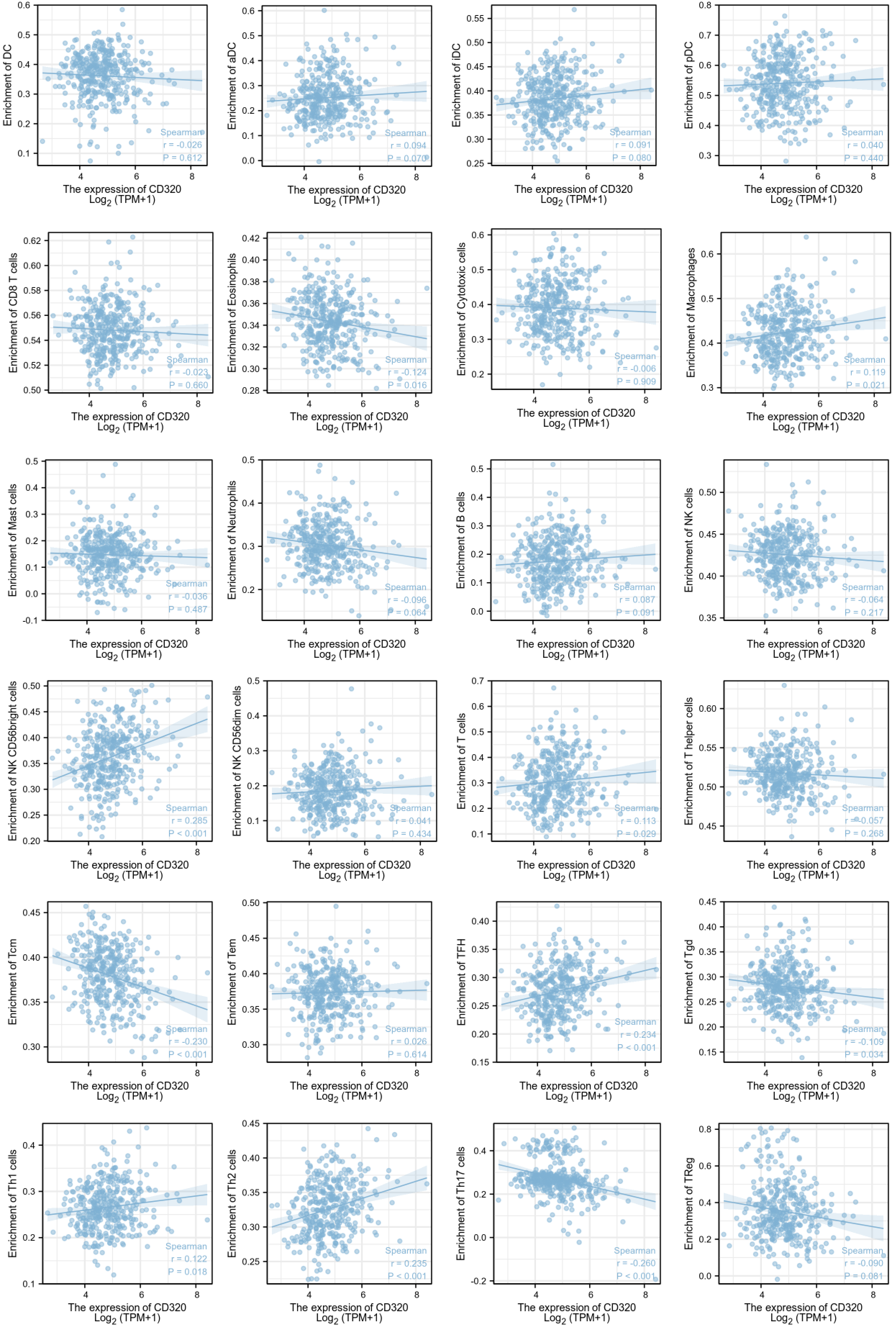

Supplement: Supplementary file 3 — Additional file 3 (TIF 9191 KB) [file 12672_2024_1122_MOESM3_ESM.tif]
